# Detection of Parkinson’s disease from EEG signals using discrete wavelet transform, different entropy measures, and machine learning techniques

**DOI:** 10.1038/s41598-022-26644-7

**Published:** 2022-12-29

**Authors:** Majid Aljalal, Saeed A. Aldosari, Marta Molinas, Khalil AlSharabi, Fahd A. Alturki

**Affiliations:** 1 Department of Electrical Engineering, King Saud University, Riyadh, Saudi Arabia; 2 Department of Engineering Cybernetics, Norwegian University of Science and Technology, Trondheim, Norway

**Keywords:** Computational neuroscience, Diseases of the nervous system, Neural ageing, Biomarkers, Diseases, Neurological disorders, Biomedical engineering, Computational science

## Abstract

Early detection of Parkinson’s disease (PD) is very important in clinical diagnosis for preventing disease development. In this study, we present efficient discrete wavelet transform (DWT)-based methods for detecting PD from health control (HC) in two cases, namely, off-and on-medication. First, the EEG signals are preprocessed to remove major artifacts before being decomposed into several EEG sub-bands (approximate and details) using DWT. The features are then extracted from the wavelet packet-derived reconstructed signals using different entropy measures, namely, log energy entropy, Shannon entropy, threshold entropy, sure entropy, and norm entropy. Several machine learning techniques are investigated to classify the resulting PD/HC features. The effects of DWT coefficients and brain regions on classification accuracy are being investigated as well. Two public datasets are used to verify the proposed methods: the SanDiego dataset (31 subjects, 93 min) and the UNM dataset (54 subjects, 54 min). The results are promising and show that four entropy measures: log energy entropy, threshold entropy, sure entropy, and modified-Shannon entropy (TShEn) lead to high classification accuracy, indicating they are good biomarkers for PD detection. With the SanDiego dataset, the classification results of off-medication PD versus HC are 99.89, 99.87, and 99.91 for accuracy, sensitivity, and specificity, respectively, using the combination of DWT + TShEn and KNN classifier. Using the same combination, the results of on-medication PD versus HC are 94.21, 93.33, and 95%. With the UNM dataset, the obtained classification accuracy is around 99.5% in both cases of off-and on-medication PD using DWT + TShEn + SVM and DWT + ThEn + KNN, respectively. The results also demonstrate the importance of all DWT coefficients and that selecting a suitable small number of EEG channels from several brain regions could improve the classification accuracy.

## Introduction

Parkinson's disease (PD) is a neurodegenerative disease that affects the elderly. According to the World Health Organization's data, this disease has claimed the lives of about 10 million individuals. Tremor, muscle stiffness, delayed movement, loss of balance, issues with walking or gait, and speech variation are all common symptoms^1,2^. Because, so far, PD is not treatable, early discovery of the disease is critical in preventing its severe consequences. The Hoehn and Yahr (HY) rating scale and the Unified Parkinson's Disease Rating Scale (UPDRS) are the most commonly used scales for assessing the severity and progression of PD. The HY scale describes PD in five stages, ranging from very few symptoms until the most hostile stage^3^.

Even while the final diagnosis is always subject to the neurologist's opinion and review, any tool that helps them contrast their diagnosis is always welcome. As a result, there is a growing demand for automated procedures that can aid in improving the accuracy of PD diagnosis. Several approaches have been presented in this regard, with the majority of them using voice signals^4–8^, gait signals^9,10^, handwriting signals^11,12^, MRI^13,14^, and only a few employing electroencephalography (EEG). EEG is considered to be one of the most important PD diagnostics tools. EEG technology can be used to capture cerebral information in a real-world context because it is reasonably inexpensive and portable^15^. In addition, EEG records brain activity faster and for a longer amount of time than other technologies. As a result, EEG analysis, along with machine learning techniques, has already been employed in the detection of several neurological conditions, including epilepsy, autism spectrum disorder, dementia and Alzheimer's disease, schizophrenia, and major depressive disorder^16–25^.

Several studies have also employed EEG signals with machine learning techniques for the detection of PD. Table 1 summarizes these studies^26–35^ with their proposed methods and corresponding results. Five out of ten PD detection studies in Table 1 proposed deep learning-based methods^26–30^. The highest classification accuracy was achieved by Khare et al.^30^. They employed the smoothed pseudo-Wigner Ville distribution (SPWVD) of EEGs with a convolutional neural network (CNN), obtaining a classification accuracy of 100%. Apart from CNN, another deep learning study by Loh et al.^29^ proposed Gabor transformation before using two-dimensional CNN, and they obtained a high classification accuracy of 99.44%. With more complex models, hybrid networks have been proposed in^27,28^. In^27^, Shah et al. developed a deep neural network architecture termed the dynamical system generated hybrid network (DGHNet). They reported that this network has a classification accuracy of 99.2%. Lee et al.^28^ proposed CNN and a recurrent neural network (RNN) with gated recurrent units (GRUs), obtaining also a classification accuracy of 99.2%. The models in^27–30^ achieved high classification accuracy, but at the expense of simplicity.

**Table 1:** Summaries of methods of previous studies and their corresponding results.

References	FE methods	Classifier(s)	Dataset	Classification accuracy (%)
^26^	–	13 layer CNN	Malaysian dataset	88.25
^27^	–	CNN + LSTM	UNM dataset	99.2
^28^	–	CNN + RNN	Own dataset	99.2
^29^	Gabor transformation + 2D-CNN		SanDiego dataset	99.44
^30^	Smoothed pseudo-Wigner Ville distribution + CNN		SanDiego dataset	100
^36^	WT + Shanono entropy	No used	Finnish dataset	No score
^31^	WT + sample entropy	Three-way decision model	Chinese dataset	92.68
^32^	Higher-order spectra (HOS)	DT,KNN,FKNN, NB,PNN,SVM	Malaysian dataset	90.6–99.6
^33^	PSD	Hyperplanes	UNM dataset	85.4
^34^	WT + statistical measures	SVM	SanDiego dataset	96.13
^35^	CSP + entropy	SVM/KNN	SanDiego + UNM	99

The other studies in Table 1^31–35^ are based on the machine learning models. Starting with Liu et al.^40^, who proposed a scheme based on discrete wavelet transform (DWT). From the resulting approximation coefficient, they employed sample entropy to compute the features. The classification of PD features was done using a three-way decision based on the optimal center constructive covering algorithm, obtaining an accuracy of 92.86%. Yuvaraj et al.^32^ employed a higher-order spectra (HOS) feature extractor to develop an automated diagnosis of PD. The bispectrum features were retrieved and their relevance assessed. Several machine learning models were used, obtaining classification accuracy ranging from 90.6 to 99.6%. Md Fahim Anjum et al.^33^ proposed linear predictive coding (LPC) to distinguish spectral EEG features of PD. They used the power spectral density (PSD) of EEG recordings. LPC was then used to extract feature vectors while classification of new subjects was done using vector projections, resulting in a classification accuracy of 85.3%. Recently, the wavelet transform was proposed by Khare et al.^34^ to decompose EEG signals into several subbands. Statistical measurements were used to extract five features from these subbands, which were then categorized using several machine learning techniques. The least square support vector machine was used to classify off-medication PD versus health control (HC) and on-medication PD versus HC, yielding accuracy of 96.13 and 97.65%, respectively. In a more recent study^35^, common spatial pattern (CSP) and entropy were combined to extract PD/HC features. Several linear and nonlinear classifiers are applied to classify the resulting PD features from normal, achieving classification accuracy of 99 and 99.41% using SVM and KNN, respectively.

It can be noted from Table 1 that at least five different datasets were used in these studies: the Finnish dataset, the Chinese dataset, Malaysian dataset, public UNM dataset, and public SanDiego dataset, making the comparison between the developed methods difficult. The table also indicates that the deep learning-based methods ^27–30^ achieve better performance than machine learning-based methods^31–35^. However, the use of machine learning models continues to attract the interest of researchers until the current time. These models are not complicated and do not require a long training period or large amounts of computer memory. However, as shown in Table 1, these models didn’t achieve competitive results (except^32^) to those of deep learning models. In machine learning-based diagnostic models, the use of efficient feature extractors is crucial to improving the diagnosis decision. As a promising method, discrete wavelet transform (DWT) has been employed in two studies ^31,34^ for PD detection, reporting a classification accuracy of 92.68 and 96.13%, respectively. In^31^, sample entropy was used to compute the features, while in^34^, statistical measurements were used. Thus, alternative DWT-based features are still required to improve PD detection. Han et al.^36^ investigated EEG abnormalities in the early stage of PD using wavelet packet and Shannon entropy. They demonstrated that EEG signals from PD patients showed significantly higher entropy over the global frequency domain, which has potential use as biomarkers of PD. However, they did not use machine learning approaches for the automatic detection.

The aim of the present study is to address these issues by presenting uncomplicated feature extraction and classification methods while maintaining high classification accuracy and validating them using two open-source datasets (the UNM and SanDiego datasets). Accordingly, simple and effective DWT-machine learning-based methods are presented for the detection of PD. The proposed methods are similar to^31,34,36^ in terms of using DWT to decompose the EEG signals and obtain approximate and details coefficients, but they differ from them in several aspects. The first aspect is that the studies^31,34,36^ computed features directly from the resulting coefficients. In the present study, it is proposed to first reconstruct the original signal from each coefficient to increase the signals’ time resolution at low frequencies, as will be discussed later. The second aspect is using different entropy measures for computing features. In other words, in addition to Shannon entropy as recommended in^36^, other types of entropy such as log energy entropy, norm entropy, sure entropy, and threshold entropy are also employed to extract PD/HC features from the reconstructed signals. The third is improving Shannon entropy by proposing to apply transformation prior, as will be discussed later. Several machine learning techniques are employed to differentiate the resulting PD features from HC ones. In addition, a greedy algorithm is employed for EEG channel selection to find the most relevant channels and investigate the possibility of achieving high classification accuracy with a smaller number of channels.

The remainder of this paper is organized as follows. "Methods" section describes the used EEG data and the following EEG signal-processing methods: preprocessing, feature extraction, classification techniques, and EEG channel selection. Results and discussion are presented in "Results and disscussion" section. "Advantages, limitations, and future studies" section includes limitations and some future study prospects. Finally, conclusions are presented in "Conclusions" section.

## Methods

**Figure 1:**
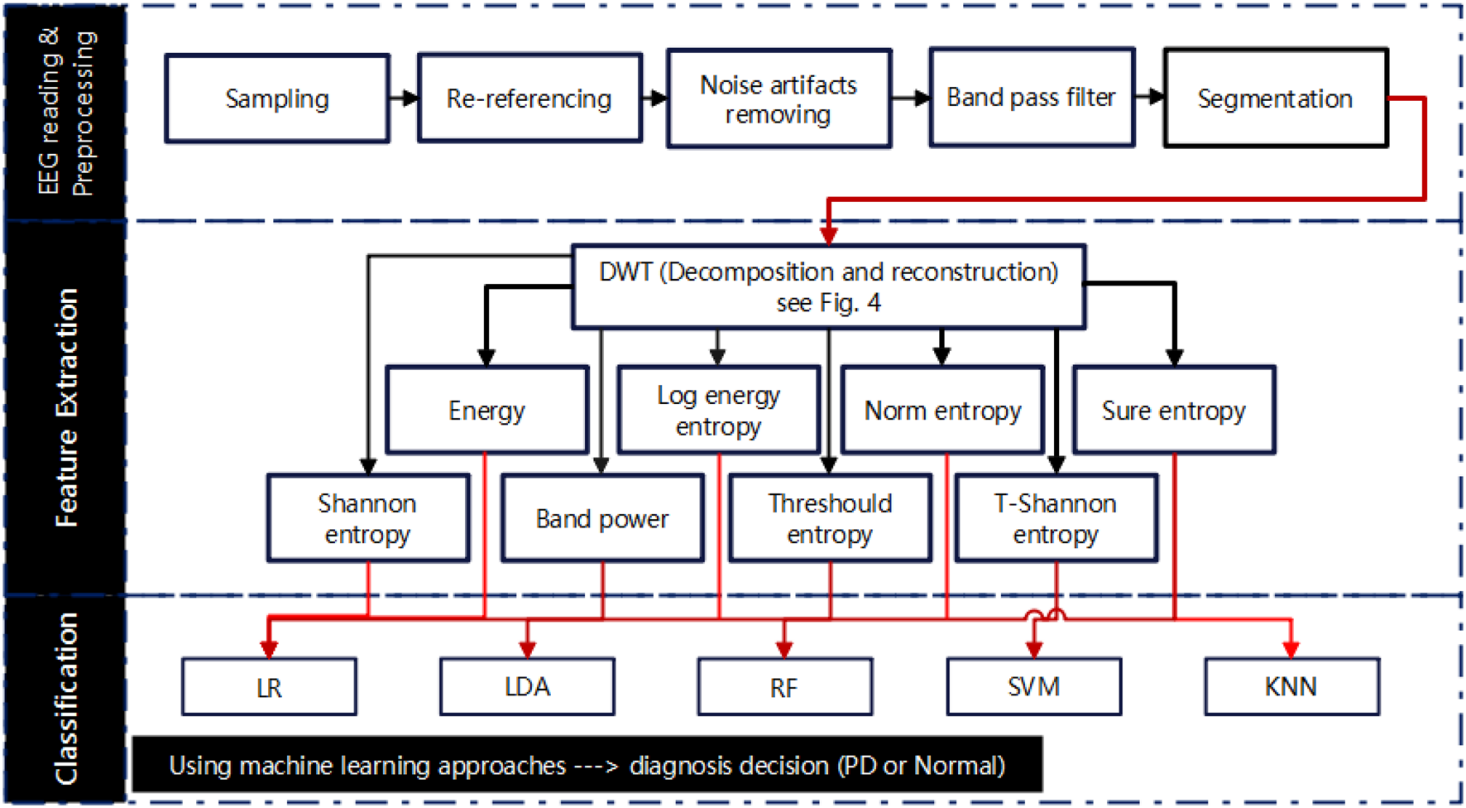
Block diagram of the proposed PD DWT-based classification methods.

This section describes the suggested methods for processing EEG signals, including data description, preprocessing, feature extraction, and classification techniques. A high-level overview of the many stages of analysis and classification of EEGs from Parkinson's patients and healthy individuals is shown in Fig. 1. The raw EEG signals are initially read, followed by a preprocessing stage to remove artifacts. In this stage, the cleaned signals are applied to a band-pass filter to locate the desired frequency region. The filtered EEG signals are then divided into equal-length segments that don't overlap. After that, the DWT algorithm is applied for decomposition and reconstruction purposes, as will be discussed later in this section. The PD/HC features are then extracted from the decomposed-reconstructed signals using a range of metrics, including band power, energy, log energy entropy, norm entropy, threshold entropy, sure entropy, and Shannon entropy. Finally, different classifiers, including logistic regression (LR), linear discriminant analysis (LDA), random forest (RF), support vector machine (SVM), and k-nearest neighbors (KNN), are employed to discriminate off/on PD features from those of healthy controls. The following subsections provide more details on each stage of the block diagram.

### Data description and pre-processing

In this study, two open-source EEG datasets are used to test the proposed approaches. The University of San Diego in California provided the first dataset^37,38^. For simplicity, this dataset is referred to as the SanDiego dataset. Table 2 includes participant demographics of patients and controls belonging to this dataset. During data collection, the subjects of this dataset were instructed to sit comfortably and unwind by focusing their gaze on a cross on a screen. This dataset consists of two groups. The first group contains EEGs of 16 healthy individuals, while the second group contains EEGs of 15 PD patients. The right-handedness, gender, age, and cognition of the PD patients were remarkably similar to those of the HC, as evaluated by the Mini-Mental State Exam (MMSE) and the North American Adult Reading Test (NAART). The disease lasted an average of 4.5–3.5 years in each patient, ranging in severity from mild to severe (Hoehn and Yahr scales II and III). On two different days, EEG data from PD patients was collected when taking medicine and when not. The healthy subjects only volunteered once. At a sampling frequency of 512 Hz, EEG data was captured for at least 3 min in a 32-channel Biosemi active EEG system. Using EEGLAB, the means for each channel were removed, and the data were all re-referenced to the common average. High-pass filtering at 0.5 Hz was used to minimize low-frequency drift. Eye blinks and movements, muscular activity, electrical noise, and other noise artefacts were manually analyzed and eliminated. This dataset's specifics, including signal capture and preprocessing, are detailed in^39^.

**Table 2:** Patient and control participant demographics (mean ± st).

	SanDiego dataset		UNM dataset	
	15 PD	16 HC	27 PD	27 HC
Age (years)	63.2 ± 8.2	63.5 ± 9.6	69.52 ± 8.66	69.52 ± 9.27
Sex	8f./7 m	9f./7 m	17f/10 m	17f./10 m
Handedness	All R	All R		
MMSE	28.9 ± 1	29.1 ± 1.1	28.68 ± 1.03	28.76 ± 1.05
NAART	46 ± 6.27	49.12 ± 7.1	45.92 ± 9.29	46.80 ± 7.64
**UPDRS**				
Off medication	41.5 ± 12.95	NA	24.80 ± 8.66	NA
On medication	33.68 ± 10.86	NA	23.36 ± 9.87	NA

**Figure 2:**
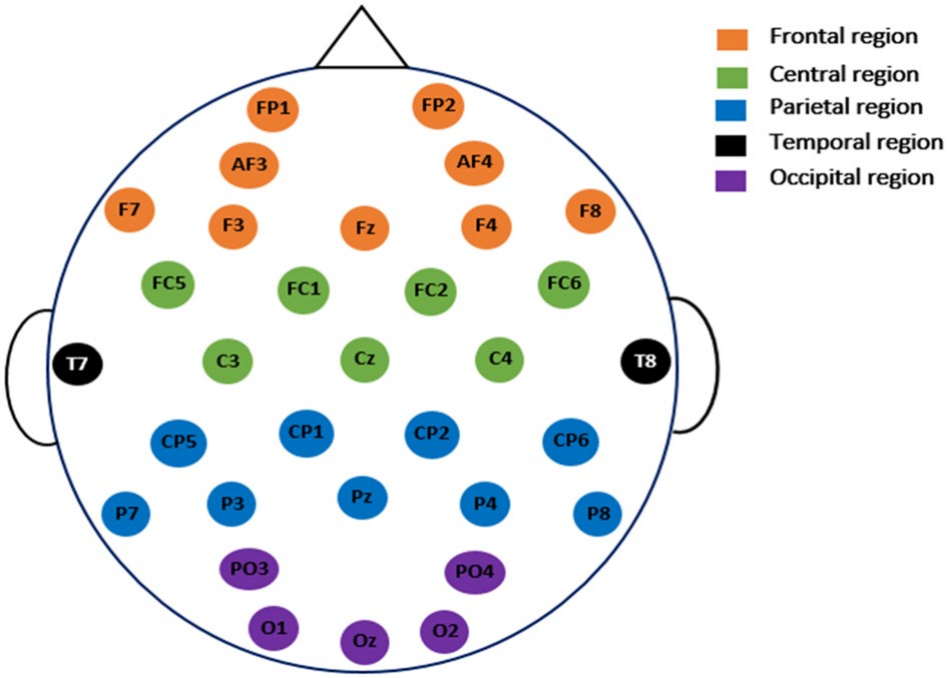
The common 32 EEG channels used in the present study^35^.

**Figure 3:**
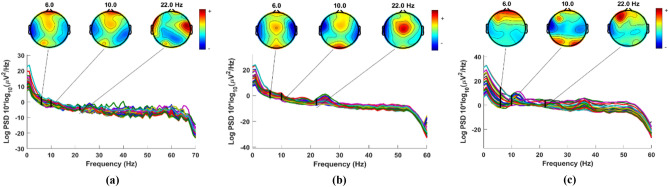
Power spectral density and electrode map for (**a**) Off-PD EEG (**b**) On-PD EEG (**c**) HC EEG^35^.

The second set of data is obtained from a study done at the University of New Mexico (UNM; Albuquerque, New Mexico). For the sake of convenience, this dataset is referred to as the UNM dataset. This dataset includes the EEGs of 27 PD patients and 27 healthy people with an equal number of genders. Table 2 also includes participant demographics of patients and controls belonging to this dataset. Seven days apart, the PD group returned to the lab twice: once while taking medication and once after a 15-h overnight withdrawal from each of their specific dopaminergic pharmaceutical prescriptions. As a result, information from 27 Parkinson's disease patients who were both on and off therapy is included in this dataset. For each patient and control, data was collected for two minutes; first, they were asked to keep their eyes closed for one minute, and then they were asked to record for another minute with their eyes open. EEG data was obtained using 64 Ag/AgCl channels at 500 Hz. With an online CPz reference and an AFz terminal grounded, the Brain Vision data gathering system is employed. The paper^40^ goes into greater detail about how the data was gathered. To analyze and evaluate the proposed techniques, we use EEG data from the 32 channels (see Fig. 2) that are available on both datasets. Figure 3 depicts electrode maps and EEG power spectral density (on a logarithmic scale) for off-PD, on-PD, and HC EEGs. The electrode map is shown for three distinct arbitrary frequencies: 6, 10, and 22 Hz. In general, the power density of the low-frequency spectrum is higher than that of the high-frequency spectrum. Different power spectral density patterns can be seen when comparing the three maps.

For further preprocessing, the EEG signals are split into segments with a size of ${\varvec{c}}{\varvec{h}}\times {\varvec{T}}$, where ${\varvec{c}}{\varvec{h}}$ is the number of channels and ${\varvec{T}}$ is the segment length in seconds. The choice of the segmentation time interval ${\varvec{T}}$ will be selected based on the length of recording of each dataset. The segmented signals are filtered using a 0.5–32 Hz fifth-order band-pass Butterworth filter. This band was selected because most of the power of PD and HC signals is concentrated in this band^33^. In addition, to remove the interference and noise caused by the electrodes and magnetic fields.

### Wavelet decomposition/reconstruction

**Figure 4:**
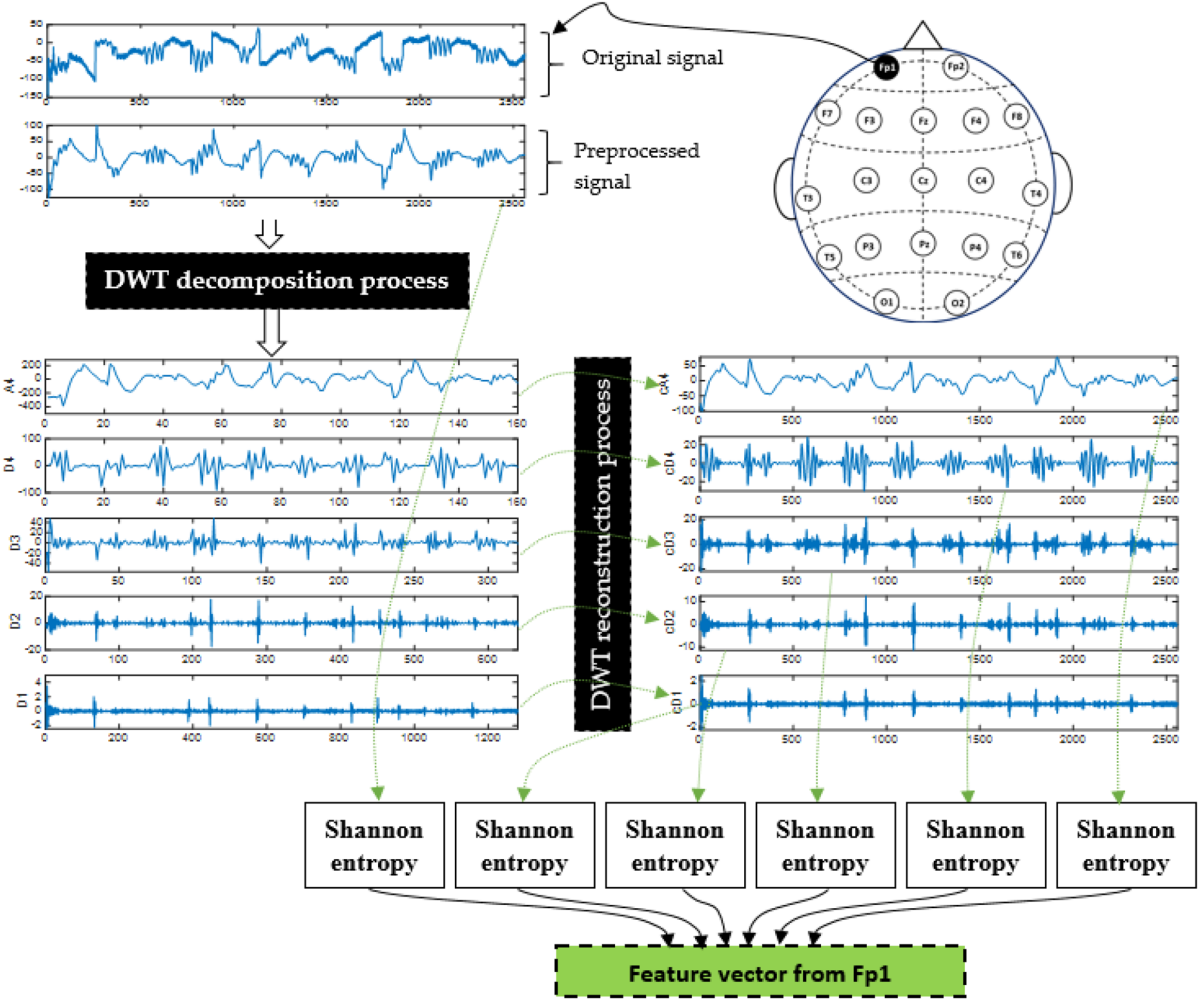
Example of feature extraction using DWT and Shannon entropy.

The discrete wavelet transform (DWT) has the ability to analyze the features of a signal in the time and frequency domains by decomposing it into a number of mutually orthogonal components using a single function called the mother wavelet^41^. The particular choice of mother function is crucial for signal analysis. Low pass and high pass filters are used in first level decomposition to produce the signal's representation as approximation (A1) and detail (D1) coefficients. The first approximation (A1) is further decomposed recursively. The number of steps (decomposition levels) is determined by the signal's major frequency components^41^. In the present study, DWT is used to decompose and then reconstruct the EEG signals into several sub-bands. The mother wavelet selected for the decomposition process is db4 as it is the most widely used in EEG signals according to the review study in^42^. Figure 4 shows the processes of decomposition and reconstruction. First, DWT decomposes each preprocessed-segmented signal from each channel into an approximate coefficient (A4) and four detail coefficients (D4, D3, D2 and D1) that correspond to 0–16 Hz, 16–32 Hz, 32–64 Hz, 64–128 Hz, and 128–256 Hz sub-bands, respectively. The coefficients with higher frequencies have good time resolution but poor frequency resolution, while the ones with the lowest frequencies have good frequency resolution but poor time resolution. Therefore, we propose reconstructing wavelet packet (WP) signals from the decomposed ones (approximate and details) to increase the signals’ time resolution at low frequencies. In other words, after obtaining all the approximation and detail coefficients (D1, D2, D3, D4 and A**4**), the signal is reconstructed from each coefficient separately as shown in Fig. 4. Consequently, five WP signals (cD1, cD2, cD3, cD4 and cA4) are produced. Because these reconstructed WP signals have good resolution in both time and frequency, it is expected that they will help produce good biomarkers for PD detection.

### Feature extraction (FE)

EEG complexity can be quantified by measuring the amount of randomness and uncertainty through non-linear methods such as entropy. Several research studies have employed entropy and machine learning techniques for the detection of brain abnormalities, demonstrating the usefulness of entropy in obtaining biomarkers for epilepsy diagnosis^43^, attention deficit hyperactivity disorder^44^, and autism diagnosis^45^. The study^36^ has also reported the usefulness of Shannon entropy as a biomarker for PD. This motivates applying machine learning techniques with different entropy measures for automatic PD detection. In the present study, in addition to Shannon entropy as recommended in^36^, other types of entropy such as log energy entropy, norm entropy, sure entropy, and threshold entropy are also investigated. Rather than directly computing entropy measures from EEG signals, as in^30–32^, we investigate computing them from each reconstructed WP signal (cD1, cD2, cD3, cD4, and cA4) to form the feature vectors shown in Fig. 4.

In addition to entropy, we also investigate energy and band power metrics, which are defined below.

#### Energy (Eng)

1 $${\mathbf{Eng}} = \mathop \sum \limits_{{{\varvec{n}} = 1}}^{{\varvec{N}}} \left| {{\varvec{S}}\left( {\varvec{n}} \right)} \right|^{2}$$

#### Band power (LBP)

2 $${\mathbf{LBP}} = \log \left[ {\frac{1}{{\varvec{N}}}\mathop \sum \limits_{{{\varvec{n}} = 1}}^{{\varvec{N}}} \left| {{\varvec{S}}\left( {\varvec{n}} \right)} \right|^{2} } \right]$$

where ${\varvec{S}}\left({\varvec{n}}\right)$ is a discrete signal (reconstructed WP signal in our case), and ${\varvec{N}}$ is signal length. If k is the number of unique values in that signal and ${{\varvec{x}}}_{{\varvec{i}}}$ is the probability frequency of the ith unique value, then the entropies are calculated as follows^46^:

#### Threshold entropy (ThEn)

3 $${\mathbf{ThEn}} = \# \left\{ {{\varvec{i}} {\mathbf{such}} {\mathbf{that}} \left| {{\varvec{x}}_{{\varvec{i}}} } \right| > {\varvec{\alpha}}} \right\}$$

According to^46^, the threshold $\boldsymbol{\alpha }$ should be less than 1. Through experimental fine-tuning, we find that setting $\boldsymbol{\alpha }=0.2$ leads to improved results in terms of accuracy.

#### Norm entropy (NoEn)

4 $${\mathbf{NoEn}} = \mathop \sum \limits_{{{\varvec{i}} = 1}}^{{\varvec{k}}} \left| {{\varvec{x}}_{{\varvec{i}}} } \right|^{{\mathbf{p}}}$$

where $\mathbf{p}$ is the power of the entropy, which must be ≥ 1. In this study, the value of p is fixed at 1.1.

#### Sure entropy (SuEn)

5 $${\mathbf{SuEn}} = {\varvec{k}} - \# \left\{ {{\varvec{i}} {\mathbf{such}} {\mathbf{that}} \left| {{\varvec{x}}_{{\varvec{i}}} } \right| \le \pounds} \right\} + \mathop \sum \limits_{{\varvec{i}}} \min \left( {{\varvec{x}}_{{\varvec{i}}}^{2} , \pounds^{2} } \right)$$

where $\mathbf{\pounds }$ is the threshold value, and generally $\mathbf{\pounds }>2$. Here, it is selected to be 3.

#### Log energy entropy (LogEn)

6 $${\mathbf{LogEn}} = \mathop \sum \limits_{{{\varvec{i}} = 1}}^{{\varvec{K}}} {\mathbf{log}}\left| {{\varvec{x}}_{{\varvec{i}}} } \right|^{2}$$

#### Shannon entropy (ShEn)

7 $${\mathbf{ShEn}} = \mathop \sum \limits_{{{\varvec{i}} = 1}}^{{\varvec{k}}} \left| {{\varvec{x}}_{{\varvec{i}}} } \right|^{2} {\mathbf{log}}\left| {{\varvec{x}}_{{\varvec{i}}} } \right|^{2}$$

#### Transformation-Shannon entropy (TShEn)

Because no good results are obtained by applying (7) to the reconstructed WP signals, we propose to transform the reconstructed signal values according to the following mapping:

8 $${\varvec{S}}_{{{\varvec{Tr}}}} = \left\{ \begin{gathered} {\varvec{T}}_{{{\varvec{max}}}} ,\quad \quad \quad \quad \quad \quad if {\varvec{S}}_{{\varvec{n}}} > 1 \hfill \\ {\varvec{T}}_{{{\varvec{max}}}} {\varvec{S}}_{{\varvec{n}}} + {\varvec{T}}_{{{\varvec{min}}}} ,\;if 0 \le {\varvec{S}}_{{\varvec{n}}} \le 1 \hfill \\ {\varvec{T}}_{{{\varvec{min}}}} ,\quad \quad \quad \quad \quad \;\;if {\varvec{S}}_{{\varvec{n}}} < 0 \hfill \\ \end{gathered} \right.$$

The effect of this transformation is two folds. First, it limits the maximum and minimum values to be within a required range [${{\varvec{T}}}_{{\varvec{m}}{\varvec{i}}{\varvec{n}}},{{\varvec{T}}}_{{\varvec{m}}{\varvec{a}}{\varvec{x}}}$]. Second, it decreases the number of unique values in the transformed signal $({{\varvec{S}}}_{{\varvec{T}}{\varvec{r}}})$. This is inspired from intensity transformation of images in order to improve the intensity in digital image processing. Here, we follow image intensity transformation and set the range to [0, 255]. We expect, in the case of WP signal processing, that this transformation is going to highlight details of reconstructed signals and reduce their randomness and complexity. After the transformation process, normalized Shannon entropy is performed on the resulting signal ${{\varvec{S}}}_{{\varvec{T}}{\varvec{r}}}$ as follows:

9 $${\mathbf{ShEn}} = \frac{1}{{\varvec{k}}}\mathop \sum \limits_{{{\varvec{i}} = 1}}^{{\varvec{k}}} \left| {{\varvec{x}}_{{\varvec{i}}} } \right|^{2} {\mathbf{log}}\left| {{\varvec{x}}_{{\varvec{i}}} } \right|^{2}$$

After extracting features using any of the metrics defined in (1)–(9), the feature vector is then formed. Since there are five reconstructed WP coefficients (cD1, cD2, cD3, cD4 and cA3), five features are extracted. A sixth element is added to the feature vector, which is obtained from the original signal segment. For ${\varvec{c}}{\varvec{h}}$ channels, the total number of features in each feature vector is $6\times {\varvec{c}}{\varvec{h}}$.

### Classification and problem formulation

In this study, we assessed the performance of several classification techniques to differentiate the PD features from the HC ones: LR, LDA, RF, SVM, and KNN. The aim is to compare between them and to determine which one provide the best results. After a manual search based on the SanDiego dataset for the parameters of each classifier, the parameters shown in Table 3 were used in the present study. More details about these classifiers can be found in^47–51^.

**Table 3:** Parameters used in the classifiers.

Classifier	Parameters
SVM	Kernel = quadratic/linear, method = ’ least square’, C = 2e−1
KNN	No. of neighbors = 3, distance = ’euclidean’, rule = ’ nearest’
RF	Learner type = ‘decision tree’, ensemble = ‘bag’, no. of learners = ’30’

### Performance evaluation

Several metrics are used to evaluate the performance of the developed models: classification accuracy, sensitivity, specificity, F-score, and receiver operating characteristic (ROC) curve. Accuracy can be calculated in terms of positives and negatives as follows:

10 $${\varvec{CA}} = \frac{{{\varvec{TP}} + {\varvec{TN}}}}{{{\varvec{TP}} + {\varvec{TN}} + {\varvec{FP}} + {\varvec{FN}}}} \times 10$$

where TP = #True Positives, TN = #True Negatives, FP = #False Positives, and FN = #False Negatives. The sensitivity and specificity are defined as follows^52^:

11 $${\varvec{Sensitivity}} = \frac{{{\varvec{TP}}}}{{{\varvec{TP}} + {\varvec{FN}}}} \times 100\%$$

12 $${\varvec{Specificity}} = \frac{{{\varvec{TN}}}}{{{\varvec{TN}} + {\varvec{FP}}}} \times 100\%$$

F-score is computed through the following formula

13 $${\varvec{F}} - {\varvec{score}} = 2 \times \frac{{{\varvec{Precision}} \times {\varvec{Sensitivity}}}}{{{\varvec{Precision}} + {\varvec{Sensitivity}}}} \times 100\%$$

where Precision is given by

14 $${\varvec{Precision}} = \frac{{{\varvec{TP}}}}{{{\varvec{TP}} + {\varvec{FP}}}} \times 100$$

The ROC curve is a graphical representation showing how the TPR (${\varvec{s}}{\varvec{e}}{\varvec{n}}{\varvec{s}}{\varvec{i}}{\varvec{t}}{\varvec{i}}{\varvec{v}}{\varvec{i}}{\varvec{t}}{\varvec{y}}$) and FPR ($1-{\varvec{s}}{\varvec{p}}{\varvec{e}}{\varvec{c}}{\varvec{i}}{\varvec{f}}{\varvec{i}}{\varvec{c}}{\varvec{i}}{\varvec{t}}{\varvec{y}}$) of a test vary in relation to one another. The area under the ROC curve (AUC) is a common metric that can be used to compare different tests. AUC values range from 0 to 1. The closer AUC is to 1 (area of unit square), the better the classifier is. Reference^53^ contains more details about ROC-AUC curves.

We employ a k-fold cross-validation (CV) technique to achieve reliable performance assessment. We used ${\varvec{k}}=10$ throughout all experiments, with 90% of the data used for training and 10% for testing. This divides the dataset into 10 equally sized subsets, one of which is used as a test set and the other nine are utilized for training^54^. The cross-validation procedure is carried out ten times. For each time, the classification performance is evaluated according to (10)–(14). Then, the results of the ten cross-validation rounds are averaged to obtain a single performance measure. In addition, leave-one-subject-out (LOSO) CV is also used, in which the data are segmented based on the subjects: one subject for test and the other remaining for training. This process is repeated until each subject has been used for test^54^.

### EEG channel selection

For channel selection, a well-known greedy algorithm called forward-elimination (FA) is employed in this study. The FA algorithm needs 32 iterations for 32 channels. In the first iteration, the classification accuracy is calculated for each single channel. The highest accuracy $MaxAcc_{1} ,$ $Max \left( {Acc_{1,2} , Acc_{1,2} , \ldots , Acc_{1,32} } \right)$, is preserved along with its corresponding channel (first local optimal), $ch\_selected_{1}$. In the second iteration, the 31 remaining channels are added, channel by channel, to $ch\_selected_{1}$ to form 31 subset of two channels. The classification accuracy is calculated for each subset. The highest accuracy $MaxAcc_{2} ,$ $Max \left( {Acc_{2,1} , Acc_{2,2} , \ldots , 31} \right)$, is preserved along with its corresponding channel subset (second local optimal), $ch\_selected_{2}$. The same operations are repeated in the remaining iterations, so that in each iteration one channel is added and the number of selected channels is increased by 1. In the 32^th^ iteration, the number of selected channels becomes 32. The final outputs of the FA algorithm are two vectors; the first vector includes the maximum accuracies, $[MaxAcc_{1} , MaxAcc_{1} , \ldots , MaxAcc_{32} ],$ while the second vector includes the corresponding subset of channels, [$ch\_selected_{1} , ch\_selected_{2} , \ldots , ch\_selected_{32}$]. The classification procedure is run 32 × 33/2 = 528 times for the 32 channels.

## Results and disscussion

### SanDiego-based results

All signals are split into 10 s segments then filtered using the BFP. Features are then extracted and classified. To present the effect of using DWT, we show the results in both scenarios. First, results in which the features are extracted by only the above measures. The other scenario is with the use of DWT in combination with the measures as described earlier. The results of the three classification problems are presented separately.

#### Off-PD vs. HC

Here, we present and discuss the results of the classification of off-medication patients versus the healthy control group, which is the main classification problem for PD detection. The number of segments obtained from off-PD is 300, while 306 segments are obtained from the HC group. The total number of segments in this case is 606. From each segment, one feature vector is extracted, where each vector contains 192 features (32 channels × 6 features). The extracted 606 × 192 feature matrix is then processed using the proposed classification techniques. Because tenfold cross-validation is employed, ten result values for each evaluation metric (accuracy, sensitivity, specificity, and F-score) are obtained. The average performance of ten values with their standard deviation is reported. Tables 4 presents classification accuracy results of the eight proposed feature metrics using the KNN classifier. The second column of the table includes the results of the proposed measures without using DWT, while the third column includes the results in the case of using DWT with those metrics. By comparing the results in the second and the third columns, the significant performance improvement in six feature measures can be seen when using DWT decomposition. The accuracy results of Shannon entropy and norm entropy have not improved in this case. The last column of Table 4 includes the results when the DWT decomposition-reconstruction is implemented before computing the features as proposed in this study. Further improvement can be seen, especially with the feature computed by log energy entropy, threshold entropy, and sure entropy. For example, in the case of the ThEn method, DWT decomposition increased the classification accuracy from 62.20 to 99.51%, and further increased to 99.72% with DWT decomposition-reconstruction. However, the results based on features computed by Shannon entropy and norm entropy have not significantly improved when DWT is used. For example, the classification accuracy obtained based on Shannon entropy is around 80% before and after using DWT. The transformation proposed in this study (Eq. 8) caused a significant improvement in Shannon entropy results, as shown in the last row of Table 4. By comparing the combination of band power, energy, and entropy measures with DWT, the results indicate that using entropy measures with DWT achieve better performance. Table 5 includes the classification performance in terms of classification accuracy, sensitivity, specificity and F-score. In order to obtain robust results, the values included in Table 5 are extensively cross-validated through ten rounds of cross-validation (10 × tenfold CV). The best performance is obtained using the DWT + TShEn method, providing 99.89% accuracy, 99.87% sensitivity, 99.91% specificity, and 99.89% F-score. Results also show that DWT + logEn, DWT + SuEn, and DWT + ThEn achieved good accuracies with low standard deviation. These four methods are further investigated next.

**Table 4:** Classification accuracy of off-PD versus HC using KNN classifier with 10 × tenfold CV.

FE methods	Without DWT	With DWT decomposition	With DWT dec. and recon
Energy	90.26 ± 4.53	96.87 ± 1.81	97.00 ± 2.34
LBP	88.96 ± 4.89	97.36 ± 3.15	97.03 ± 2.08
LogEn	91.58 ± 3.07	96.87 ± 2.12	97.94 ± 1.71
ShEn	81.67 ± 3.47	80.37 ± 5.32	80.33 ± 4.61
ThEn	62.20 ± 8.45	99.51 ± 1.11	99.72 ± 0.66
SuEn	86.96 ± 3.98	98.68 ± 1.05	99.66 ± 0.76
NoEn	87.14 ± 3.98	87.13 ± 5.47	89.98 ± 4.07
TShEn	70.77 ± 6.65	98.36 ± 1.35	99.89 ± 0.31

**Table 5:** Classification results of off-PD versus HC using KNN classifier (with DWT dec/recon).

FE methods	Accuracy (%)	Sensitivity (%)	Specificity (%)	F-score (%)
	Mean ± st	Mean ± st	Mean ± st	Mean ± st
DWT + Eng	97.00 ± 2.34	96.33 ± 3.48	97.89 ± 2.86	97.00 ± 2.32
DWT + LBP	97.03 ± 2.08	96.31 ± 3.24	97.95 ± 2.56	97.04 ± 2.06
DWT + LogEn	**97.94 ± 1.71**	**97.35 ± 2.77**	**98.72 ± 2.07**	**97.97 ± 1.80**
DWT + ShEn	80.33 ± 4.61	78.96 ± 6.05	82.59 ± 5.60	80.66 ± 4.41
DWT + ThEn	**99.72 ± 0.66**	**99.52 ± 1.21**	**99.92 ± 0.23**	**99.72 ± 0.68**
DWT + SuEn	**99.66 ± 0.76**	**99.37 ± 1.43**	**99.97 ± 0.11**	**99.66 ± 0.75**
DWT + NoEn	89.98 ± 4.07	89.22 ± 5.25	91.22 ± 4.85	**90.01 ± 4.03**
DWT + TShEn	**99.89 ± 0.30**	**99.87 ± 0.37**	**99.91 ± 0.25**	**99.89 ± 0.31**

Significant values are in [bold].

**Figure 5:**
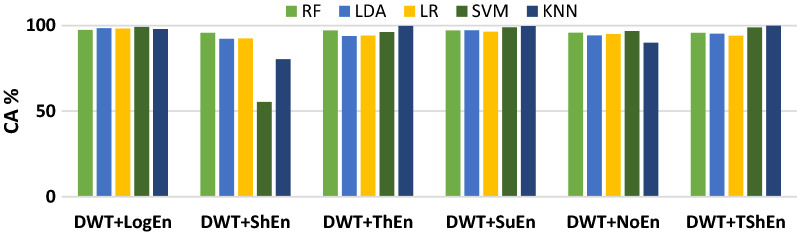
Average classification accuracy (off-PD vs. HC) using RF, LDA, LR, SVM, and KNN.

**Figure 6:**
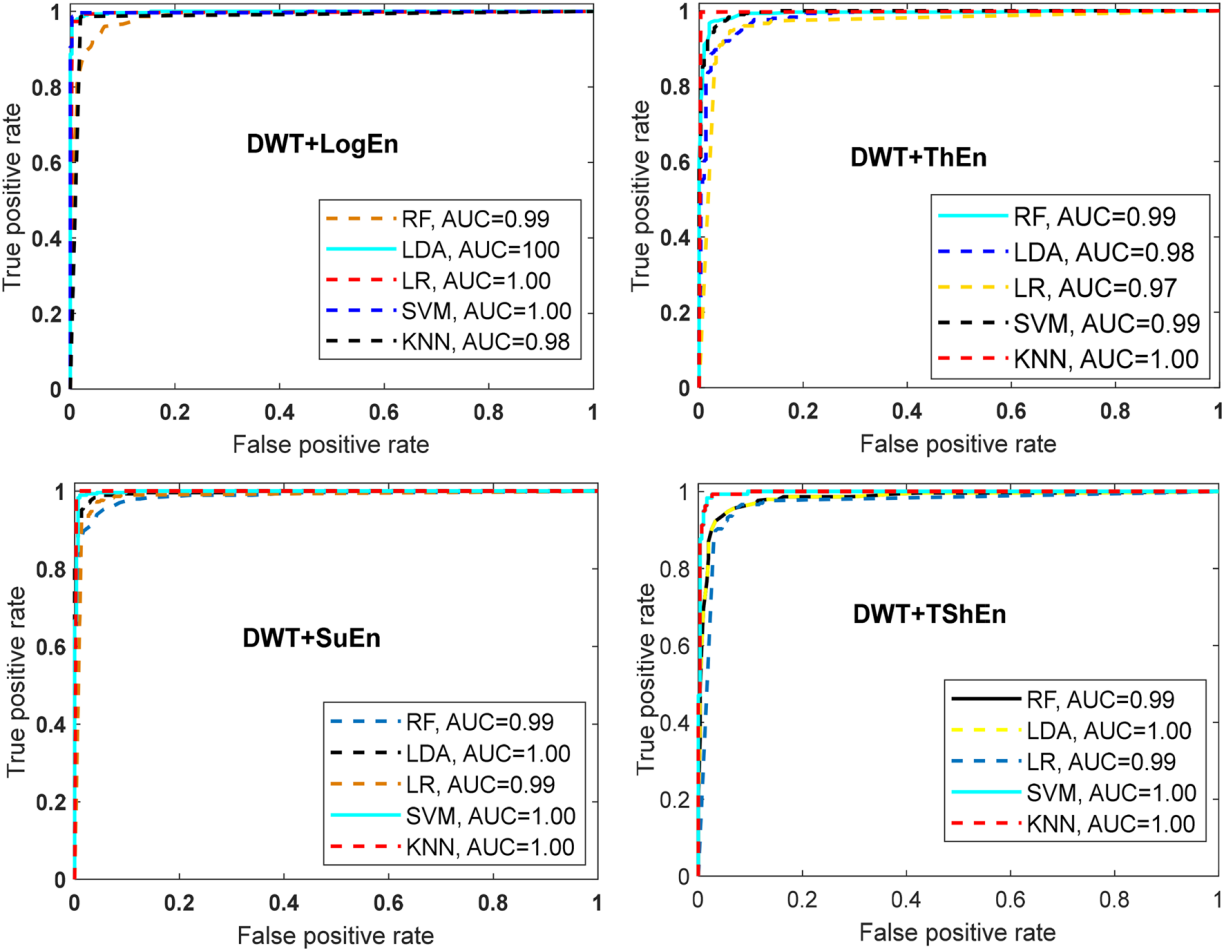
ROC-AUC of off-PD versus HC classification based on features extracted from four methods.

**Figure 7:**
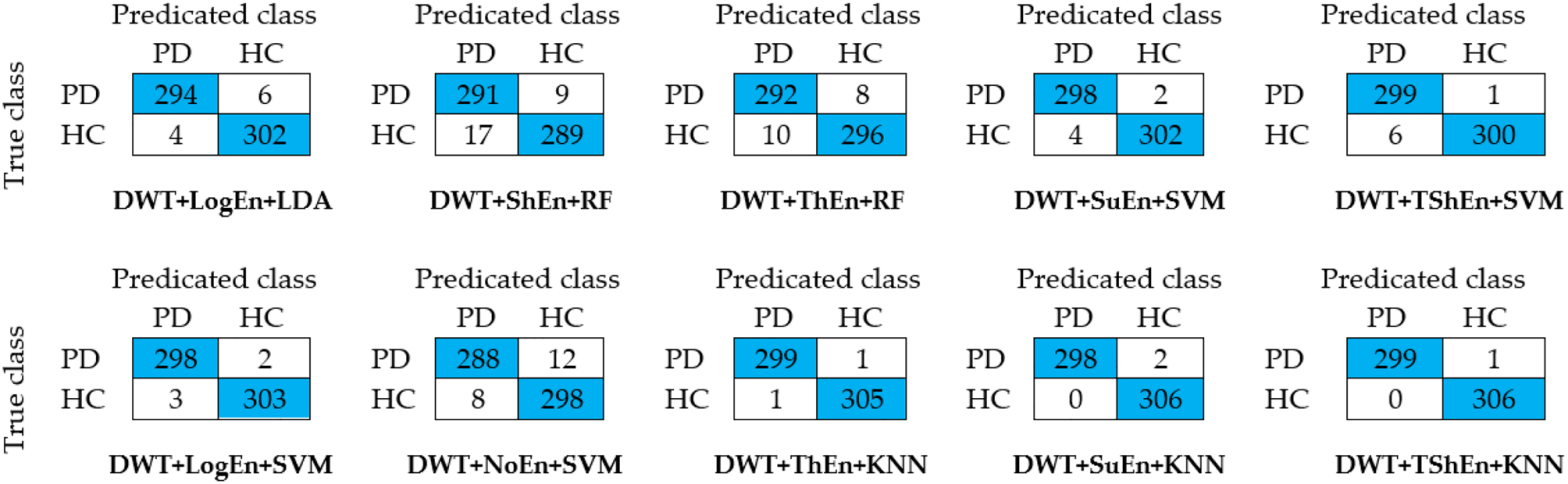
Confusion matrices for selected methods (off-PD vs. HC).

Figure 5 presents the classification accuracy results using RF, LDA, LR, SVM, and KNN machine learning approaches with the six entropy metrics. As shown in Fig. 7, all classifiers perform equally well in classifying the features extracted by the WT + logEn method, indicating that this method works well regardless of the classifier used. By comparing the classifiers, it can be noted that the KNN and quadratic-SVM classifiers achieve the best results with the most FE methods, while LR and LDA achieve the worst. This is because the resulting features are obtained by non-linear methods, and therefore efficient non-linear classifiers are needed to obtain high accuracy. The authors of ^32^ also investigated several linear and nonlinear classifiers to classify HOS-based features. The author reported that nonlinear classifiers perform well as EEG signals are nonlinear in nature. Returning to Fig. 5, it is reported that the three highest classification accuracy scores (99.89, 99.72, and 99.66%) are achieved by the KNN classifier when the features are extracted by DWT + TShEn, DWT + ThEn, and DWT + SuEn, respectively. SVM also performs well, especially with DWT + LogEn, DWT + TShEn, and DWT + SuEn, with accuracy values of 99.15, 98.95, and 98.83%, respectively. For more investigation, four FE methods that achieve the best performance are selected for obtaining ROC curves along with AUC of each classifier (see Fig. 6). Figure 5 shows ROC curves along with the AUC of each classifier. Figure 6 also shows that KNN and SVM achieve the best performance.

In order to investigate the ability of the proposed methods to identify PD/HC, the confusion matrices have been presented in Fig. 7 for ten complete methods. It can be seen from the matrices that there is no big differences between sensitivity and specificity in most methods. Fewer mistakes were made by KNN when combining with DWT + ThEn, DWT + SuEn, and DWT + TShEn FE methods. For example, in the case of DWT + TShEn + KNN, of the 300 vectors belonging to PD, only one was classified as normal, while all vectors belonging to normal are correctly classified.

In order to investigate the ability of the proposed methods to identify PD/HC, the confusion matrices have been presented in Fig. 7 for ten complete methods. It can be seen from the matrices that there is no big differences between sensitivity and specificity in most methods. Fewer mistakes were made by KNN when combining with DWT + ThEn, DWT + SuEn, and DWT + TShEn FE methods. For example, in the case of DWT + TShEn + KNN, of the 300 vectors belonging to PD, only one was classified as normal, while all vectors belonging to normal are correctly classified.

##### Investigation of wavelet coefficients

**Figure 8:**
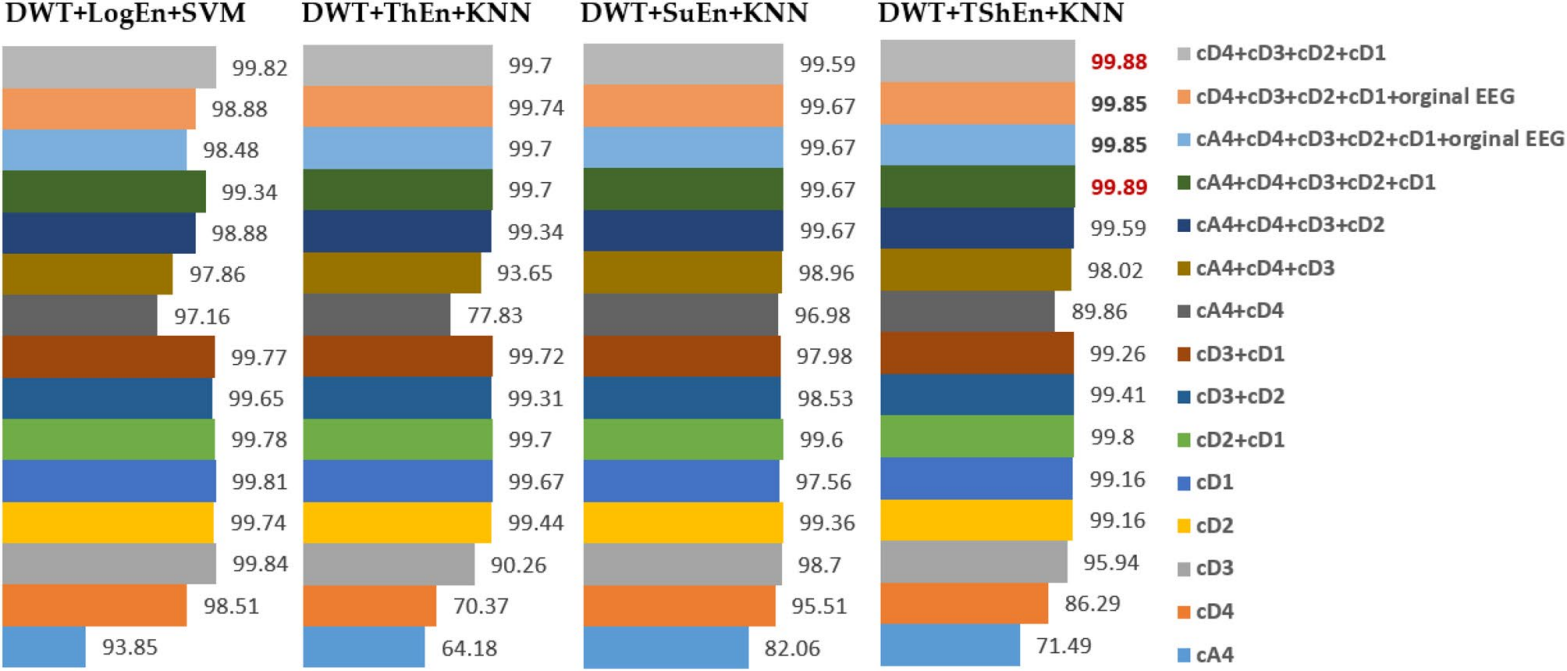
The effect of combination of wavelet coefficients on the classification performance (off-PD vs. HC).

Here, we investigate the effect of wavelet coefficients (cA4, CD4, cD3, cD2, cD1) on the classification performance with four FE methods: DWT + LogEn, DWT + ThEn, DWT + SuEn, and DWT + TShEn. The purpose is to find out which coefficients contain the most relevant information for off-medication PD detection. Figure 8 shows the results in terms of average classification obtained from each coefficient separately as well as from the combined coefficient. The obtained accuracy scores have also been cross-validated through ten rounds of cross-validation (10 × tenfold CV). Looking at the first five rows in the figure and comparing the results obtained from each coefficient separately, it can be seen that the features extracted from cD1, cD2, and cD3 are more accurately classified than those extracted from other coefficients. This indicates that the higher frequency bands contain important information to be used for PD detection, as confirmed in^36^. The author of^36^ combined WT and Shannon entropy (WPE) to characterize EEG signals in different frequency bands between the PD and HC groups. They found that WPE in the γ -band of PD patients was higher than that of HC, while WPE in the δ, θ, α, and β bands were all lower^36^. They also reported that these changes in EEG dynamics may represent early signs of cortical dysfunction, which have potential use as biomarkers of PD in the early stages. In addition, the authors of^27^ showed that there is strong synchronization between the amplitude of higher frequency components and the phase of β components for PD patients. Figure 8 also shows that the combination of cD1 with cD2 or cD2 with cD3 leads to higher accuracy. However, the highest accuracy scores are obtained when all features extracted from (cD1, cD2, cD3, cD4, and cA4) are combined, indicating that all coefficients may represent signs for PD detection.

##### Investigation of channels

Figure 2 illustrates the distribution of the 32 used channels into five regions: frontal (F), central (C), parietal (P), temporal (T), and occipital (O). Here, we investigate which regions/channels contain important information for classifying EEGs of PD versus normal ones. To this end, a region-based classification is performed. In the present investigation, the features extracted from cD1, cD2, cD3, cD4, and cA4 coefficients are included. Table 6 shows that the features extracted from F region channels are classified with higher accuracy. The P region and C region channels come in second and third place, respectively. Obtaining the highest classification accuracy from the F region supports the results of^40^, which showed that the most informative features were predominantly in the frontal area. Because PD is related to motor decline, the authors of^27^ investigated the use of individual component analysis (ICA) to demonstrate that the strengthened synchronizations can be cumulatively collected from EEG channels over the motor region (C) of the brain. They used this information and selected 12 EEG channels belonging to the central and centro-parietal regions for PD classification. Our results support their finding that the P and C regions contain relevant information for PD identification. In the present investigation (Table 6), features from multiple regions are combined (F + C, F + P, C + P, and F + C + P), which provided a significant accuracy improvement, especially for the last combination. These results indicate that the important information for off-PD detection is not limited to only one region. Therefore, two additional cases are considered for which the classification was based on a smaller number of channels distributed over different regions. The last two rows of Table 6 contain the channels used and their corresponding results. For example, when using only four channels (Fz, Cz, Pz, and Oz), the classification accuracy reaches 95.36% for the DWT + TShEn + KNN method. The classification accuracy becomes 98.60% when the number of channels is increased to 8. This finding supports the results of^33^, which compared 62 EEG channels with individual best performances. This study^33^ reported that the central electrodes had good accuracy but the most effective channels were from 10 channels distributed over different regions. So, here comes the importance of applying a method for selecting EEG channels to select the group of channels that provide the best results. Table 7 includes the classification accuracy scores based on the EEG channels selected using the forward-addition method that was already discussed. It can be seen from the table that a small suitable number of channels could achieve high accuracy. For example, in the case of DWT + ThEn + KNN and DWT + TShEn + KNN, selecting a combination of 10 channels from 32, from different regions, achieves accuracies of 99.44 and 99.72%, respectively.

**Table 6:** Investigation of channels used on the classification performance (off-PD vs. HC).

Region (or channels)	#feature/vector	FE Method + classifier				
		DWT + LogEn + KNN	DWT + LogEn + SVM	DWT + ThEn + KNN	DWT + SuEn + KNN	DWT + TShEn + KNN
Frontal region (F): Fp1, AF3, F7, F4, F8, AF4, Fp2, Fz	40	95.31 ± 2.68	96.95 ± 1.94	97.64 ± 1.82	97.92 ± 1.85	97.37 ± 1.94
Central region (C): FC1, FC5, C3, C4, FC6, FC2, Cz	35	95.75 ± 2.76	95.21 ± 2.82	95.63 ± 2.98	95.51 ± 2.76	94.32 ± 2.84
Parietal region (P): CP1, P4, P7, P3, Pz, P4, P8, CP6, CP2	45	96.95 ± 2.25	97.46 ± 2.08	97.13 ± 1.95	96.68 ± 2.43	96.60 ± 2.34
Temporal region (T): T7,T8	10	86.72 ± 3.86	75.09 ± 5.39	76.74 ± 6.04	83.15 ± 4.90	76.72 ± 5.42
Occipital region (O): PO3, O1, Oz, O2, PO4	25	93.76 ± 3.09	91.90 ± 3.53	94.25 ± 3.02	92.94 ± 3.09	89.42 ± 3.59
F + C	75	97.38 ± 2.10	97.92 ± 1.95	98.81 ± 1.23	98.88 ± 1.45	98.78 ± 1.40
F + P	85	97.57 ± 1.95	98.78 ± 1.36	99.13 ± 1.18	99.06 ± 1.38	98.81 ± 1.38
C + P	80	97.16 ± 2.01	98.04 ± 1.86	98.68 ± 1.40	98.55 ± 1.47	98.18 ± 1.65
F + C + P	120	97.79 ± 1.91	98.84 ± 1.40	99.34 ± 1.06	99.09 ± 1.27	99.35 ± 1.09
Fz, Cz, Pz, Oz	20	97.11 ± 1.90	95.05 ± 2.56	95.59 ± 2.74	96.87 ± 2.32	95.63 ± 2.46
F7, F8, C3, C4, P7, Pz, P8, Oz	40	97.49 ± 2.04	97.18 ± 2.04	97.99 ± 1.71	98.74 ± 1.45	98.60 ± 1.33

**Table 7:** The classification accuracy based on subsets with 1–10 channels selected by FA algorithm.

No. of selected channels by FA	#Feature/vector	FE Method + classifier				
		DWT + LogEn + KNN	DWT + LogEn + SVM	DWT + ThEn + KNN	DWT + SuEn + KNN	DWT + TShEn + KNN
1	5	88.29 ± 4.31	76.00 ± 5.36	80.24 ± 4.69	79.90 ± 4.75	79.04 ± 4.60
2	10	94.93 ± 2.82	91.82 ± 3.60	89.97 ± 3.86	91.52 ± 3.27	92.05 ± 3.36
3	15	96.63 ± 2.15	96.62 ± 2.50	95.15 ± 3.02	95.85 ± 2.36	96.54 ± 2.23
4	20	97.70 ± 1.90	96.86 ± 1.96	97.33 ± 2.14	97.39 ± 1.94	97.74 ± 2.04
5	25	97.76 ± 1.69	97.29 ± 2.11	98.27 ± 1.79	98.22 ± 1.57	98.05 ± 1.92
6	30	–	98.46 ± 1.59	98.70 ± 1.79	98.83 ± 1.48	98.58 ± 1.61
7	35	98.27 ± 1.93	98.98 ± 1.37	99.01 ± 1.17	99.13 ± 1.23	98.70 ± 1.53
8	40	98.19 ± 1.68	–	99.21 ± 1.05	99.44 ± 1.02	99.19 ± 1.09
9	45	–	–	–	99.47 ± 1.03	–
10	50	–	99.34 ± 1.12	99.44 ± 0.87	–	99.72 ± 0.70

Our proposed methods are compared with existing state-of-the-art techniques to assess their effectiveness for off-PD versus HC classification. From the previous studies summarized in Table 1, we focus on studies that used the same dataset^29,30,34,35^. In^29^, first, the Gabor transformation was used to convert the EEG signals to spectrograms. After that, these spectrograms were used to train a two-dimensional convolutional neural network (2D-CNN) model, obtaining a high classification accuracy of 99.44%. In^30^, Khare et al. employed the smoothed pseudo-Wigner Ville distribution (SPWVD) of EEGs with CNN, obtaining a classification accuracy of 99.84%. In^34^, Khare et al. also proposed the wavelet transform to decompose EEG signals into several subbands. After that, statistical measurements were used to extract five features from these subbands, obtaining an accuracy of 96.13% using the least square SVM. The authors of^35^ proposed a combination of common spatial pattern and entropy, obtaining an accuracy of 99.41% using the KNN classifier. The present study used the discrete wavelet transform with threshold entropy, sure entropy, or proposed T-Shannon entropy, achieving accuracy scores of 99.72, 99.66, and 99.89%, respectively (see Table 5). These results are superior to the results of^29,34,35^, and close to the result reported in^30^.

#### On-PD vs. HC

**Figure 9:**
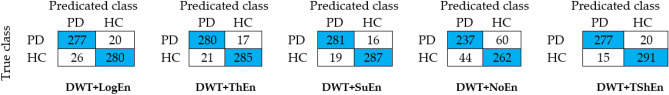
Confusion matrices for KNN classifier and different FE methods (on-PD vs. HC).

**Figure 10:**
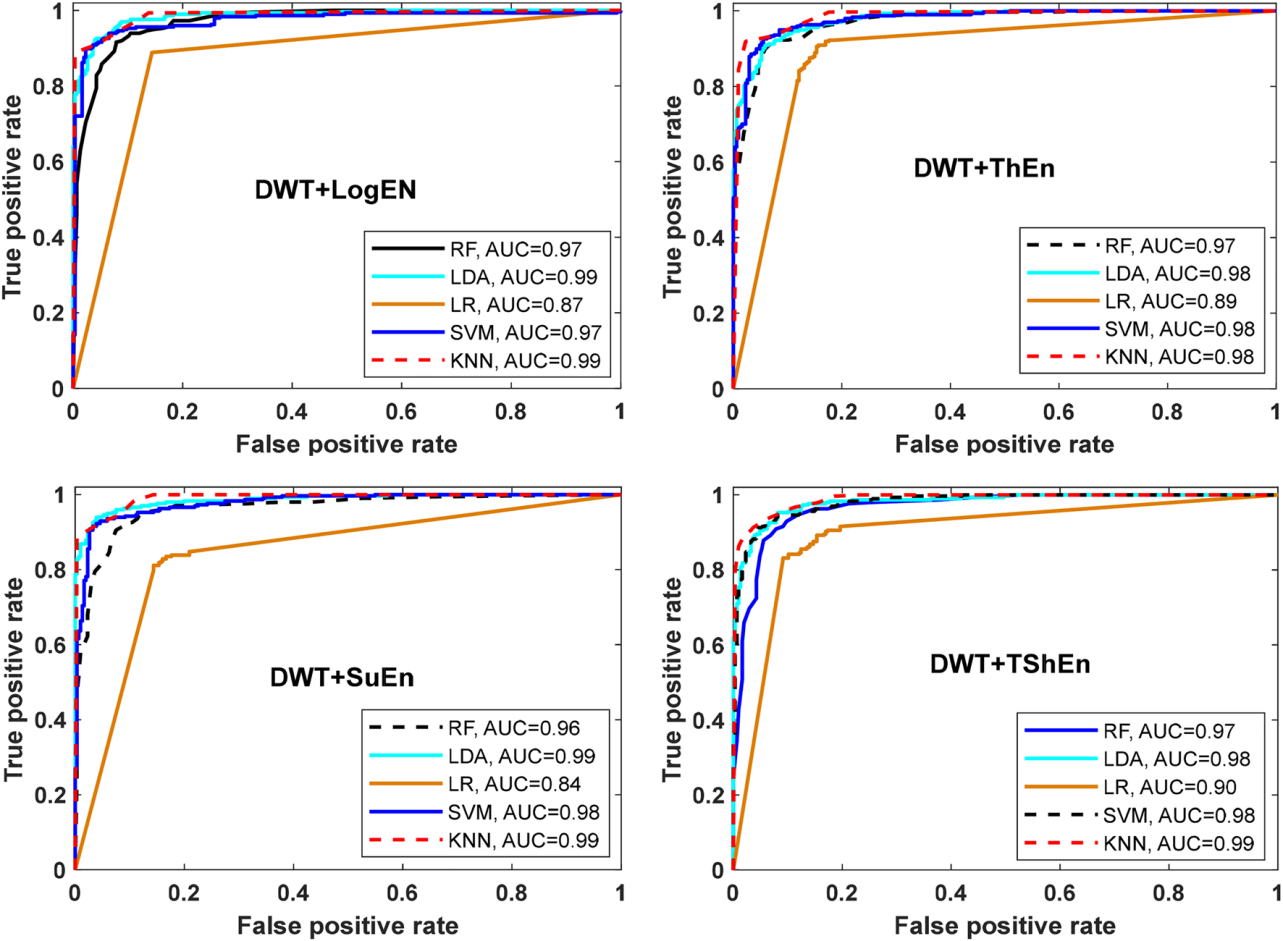
ROC-AUC of off- PD versus HC classification based on features extracted from four feature extraction methods.

This classification is useful to study the effects of levodopa medicine on PD patients. The number of feature vectors in this case is 603, of which 297 vectors come from on-PD while 306 are from HC. Table 8 shows that the DWT + TShEn and DWT + SuEn methods achieve the best performance, with similar scores of accuracy but different scores of sensitivity and specificity. The DWT + SuEn method achieves the highest sensitivity of 94.58%, while the DWT + TShEn method achieves the highest specificity of 95.01%. In the case of DWT + SuEn, Fig. 9 shows that of the 297 vectors belonging to PD, 16 were classified as normal and the remaining vectors were correctly classified. In the case of DWT + TShEn, of the 306 vectors belonging to HC, 15 were miss-classified and 291 were correctly classified. Table 8 and Fig. 9 also show that DWT + ThEn, DWT + SuEn, and DWT + LogEn methods provide superior classification performance compared to other addressed methods. By comparing the combination of band power, energy, and entropy measures with DWT, like in off-PD detection, the results indicate that using entropy metrics with DWT achieve better performance. Figure 10 shows ROC curves along with AUC for the four best FE methods with the four classifiers. The KNN classifier has the best performance, while LR achieves the worst.

**Table 8:** Classification results of on-PD versus HC using KNN classifier.

FE methods	Accuracy (%)	Sensitivity (%)	Specificity (%)	F-score (%)
	Mean ± st	Mean ± st	Mean ± st	Mean ± st
DWT + Eng	91.37 ± 3.40	90.69 ± 4.74	92.60 ± 4.97	91.32 ± 3.48
DWT + LBP	90.87 ± 3.29	89.89 ± 3.97	92.18 ± 4.68	90.79 ± 3.45
DWT + LogEn	**92.22 ± 2.43**	**93.34 ± 3.92**	**91.60 ± 3.98**	**91.97 ± 2.56**
DWT + ShEn	76.77 ± 4.56	75.71 ± 5.78	78.64 ± 6.20	76.79 ± 4.84
DWT + ThEn	**93.71 ± 3.74**	**94.00 ± 3.63**	**93.14 ± 3.89**	**93.94 ± 3.34**
DWT + SuEn	**94.20 ± 2.23**	**94.58 ± 4.67**	**93.79 ± 4.24**	**93.95 ± 3.15**
DWT + NoEn	82.41 ± 4.05	79.91 ± 4.68	85.75 ± 5.46	82.79 ± 4.09
DWT + TShEn	**94.21 ± 2.71**	**93.33 ± 2.11**	**95.01 ± 2.00**	**94.40 ± 1.63**

Significant values are in [bold].

When off-PD and HC classification results from the previous section are compared to the results of on-PD and HC classification from this section, it is clear that the proposed approaches perform better in the first classification problem. The highest classification accuracy achieved for off-PD/HC classification was 99.89%, while the highest accuracy in the case of on-PD/HC classification was 94.21%. This was expected because each PD patient's EEG recordings showed a different response to dopaminergic treatment. The dataset's creators, Swann et al. ^38^, also noted that they saw elevated phase-amplitude coupling in PD patients who weren't taking medication; this occurrence was noted in 14 out of 15 of their PD patients.

The studies^29,30,34,35^ also employed their models for on-PD versus HC classification, obtaining high accuracy scores. The highest accuracy of 100% was achieved by the model proposed in^30^, while the model in^29^ came in the second rank with accuracy of 98.84%. The models developed in^29,30^ achieved high classification scores, outperforming our results, but at the expense of simplicity. The methods proposed in^34,35^ achieved accuracy scores of 97.65 and 95.76%, respectively, which are not far from our results.

#### Off-PD vs. On-PD

The aim of this classification problem is to test the effectiveness of the methods proposed in the present study. Table 9 shows that features extracted from channels placed in the F region were also classified more accurately than those from other regions. However, when features are taken from suitable channels distributed across different regions, the accuracy improves. For example, the accuracies resulting from only four channels selected by FA are 96.90, 97.22, 97.09, and 96.70% when the features are extracted by DWT + LogEn, DWT + ThEn, DWT + SuEn, and DWT + TShEn, respectively. Furthermore, the results show that eight suitable channels selected by FA produce higher accuracy than that obtained from all channels. This indicates that information about PD is not confined to one region. Compared with those who used the same dataset, only two studies^29,35^ investigated the off-PD versus on-PD classification problem. The accuracy scores reported by these two studies were 92.60 and 97.52%, respectively. Our findings are superior to these results in both cases: with the use of all channels or with only the eight channels selected by FA.

**Table 9:** Classification results of off-PD versus on-PD using KNN classifier.

Region (or channels)	FE Methods			
	DWT + LogEn	DWT + ThEn	DWT + SuEn	DWT + TShEn
Frontal region (F): Fp1, AF3, F7, F4, F8, AF4, Fp2, Fz	95.81 ± 2.53	96.31 ± 2.72	94.47 ± 3.27	94.97 ± 3.80
Central region (C): FC1, FC5, C3, C4, FC6, FC2, Cz	94.46 ± 2.41	93.81 ± 3.76	94.30 ± 2.42	94.81 ± 2.01
Parietal region (P): CP1, P4, P7, P3, Pz, P4, P8, CP6, CP2	92.31 ± 4.31	93.98 ± 3.16	92.96 ± 1.91	92.64 ± 3.43
Occipital region (O): PO3, O1, Oz, O2, PO4	91.62 ± 3.64	87.62 ± 5.31	89.28 ± 4.99	86.09 ± 3.88
Four Channels selected by FA	96.90 ± 2.20	97.22 ± 2.10	97.09 ± 2.32	96.70 ± 2.13
Eight Channels selected by FA	98.38 ± 1.74	98.33 ± 1.79	98.56 ± 1.47	98.84 ± 1.40
All channels (32)	97.82 ± 1.94	97.65 ± 2.14	97.99 ± 1.91	97.49 ± 1.98

### UNM-based results

In this section, the proposed methods are assessed and validated using the UNM dataset. As described in "Data description and pre-processing" section, the UNM dataset is collected using 68 channels. To make the comparison fair, only the same channels that were used to record the SanDiego dataset are used in the present investigation (see Fig. 2). Because the features extracted from cD1, cD2, cD3, cD4, and cA4 coefficients led to the best performance as investigated with the SanDiego dataset, the same coefficients are also included in the present investigation. In addition, the same classifiers parameters are also used. Since the length of recording for each state is only one minute, all signals in this dataset are divided into segments with a length of 2 s to increase the number of feature vectors. Four FE methods: DWT + LogEn, DWT + ThEn, DWT + SuEn, and DWT + TShEn, are used with two states: open-eyes and close-eyes. Tables 10 and Table 11 show the number of features vectors in each case and the classification results using KNN and SVM classifiers. The results in the two tables show the classification accuracy scores are similar for both cases: open-eyes and close-eyes states. In the cases of off-PD versus HC and off-PD versus on-PD classification problems, the DWT + TShEn + SVM method achieved the highest accuracy scores of 99.51 and 99.39% for the two problems, respectively. The DWT + ThEn + KNN achieved the highest accuracy of 99.52% for on-PD versus HC. These accuracy scores are obtained with open-eyes state.

**Table 10:** Classification results using KNN classifier (UNM dataset).

	Classification accuracy (mean ± st)					
	Off-PD versus HC		On-PD versus HC		Off-PD versus On-PD	
	Open eyes	Close eyes	Open eyes	Close eyes	Open eyes	Close eyes
**#feat. vectors**	810 PD + 810 HC	810 PD + 783 HC	840 PD + 810 HC	840 PD + 783 HC	1650	1650
DWT + LogEn	98.64 ± 0.81	99.06 ± 0.61	98.91 ± 1.21	98.71 ± 1.31	98.73 ± 0.92	98.36 ± 1.18
DWT + ThEn	99.14 ± 0.52	99.43 ± 0.86	99.52 ± 0.56	99.20 ± 0.65	99.27 ± 0.75	99.21 ± 0.70
DWT + SuEn	97.53 ± 1.52	96.17 ± 0.96	96.73 ± 1.52	95.75 ± 2.00	97.09 ± 1.14	94.91 ± 1.38
DWT + TShEn	99.14 ± 0.93	99.18 ± 0.52	99.21 ± 0.64	98.89 ± 0.57	99.27 ± 0.69	98.73 ± 0.83

**Table 11:** Classification results using SVM classifier (UNM dataset).

	Classification accuracy (mean ± st)					
	Off-PD versus HC		On-PD versus HC		Off-PD versus On-PD	
	Open eyes	Close eyes	Open eyes	Close eyes	Open eyes	Close eyes
**#feat. vectors**	810 PD + 810 HC	810 PD + 783 HC	840 PD + 810 HC	840 PD + 783 HC	1650	1650
DWT + LogEn	99.14 ± 0.93	98.31 ± 0.73	99.58 ± 0.57	98.46 ± 0.52	99.21 ± 0.64	98.18 ± 1.18
DWT + ThEn	96.91 ± 1.13	95.80 ± 1.29	96.85 ± 1.98	95.87 ± 2.00	95.70 ± 1.16	95.52 ± 1.83
DWT + SuEn	92.84 ± 1.43	93.41 ± 1.94	93.15 ± 2.25	93.28 ± 1.65	93.15 ± 2.04	93.45 ± 2.13
DWT + TShEn	99.51 ± 0.64	98.62 ± 0.88	99.39 ± 0.64	99.45 ± 0.61	99.39 ± 0.81	98.79 ± 0.90

These results are compared with the results of those studies that used the same dataset^27,33,35^. In^27^, a hybrid deep neural network architecture based on a combination of CNN and long-term memory (LSTM) was developed. The authors of^33^ proposed LPC to distinguish spectral EEG features of PD. They used the PSD of EEG recordings. LPC was then used to extract feature vectors while classification of new subjects was done using vector projections. In^35^, the authors combined CSP and entropy to extract PD/HC features and then classified them using KNN and SVM classifiers. In^27^, Shah et al. considered only off-PD versus on-PD classification problem, obtaining an accuracy of 99.2%. Anjum et al.^33^ reported that there was no statistically significant effect between levodopa-on and levodopa-off sessions. With their proposed method, an accuracy of 85.3% was obtained. In^35^, the authors investigated three classification problems: off-PD versus HC; on-PD versus HC; and off-PD versus on-PD. Their proposed methods achieved accuracy scores of 98.81, 98.77, and 98.73 for the first, second, and third problems, respectively. Compared with the results of^27,33,35^, our results outperform them in the three classification problems.

In addition to the k-fold CV, the leave-one-subject-out (LOSO) CV technique is also used to validate our methods with the UNM dataset (54 subjects). One of the 54 subjects is used for test, while the remaining 53 are used for training in this technique. This process is repeated 54 times (through 54 iterations) by changing the test subject and training subjects. The classification accuracy is computed at each iteration, and the average accuracy is calculated over the 54 iterations. Table 12 shows the classification accuracy results of on-PD versus HC with the use of FA algorithm for channel selection. The results indicate that the validation by LOSO presented accuracy scores less than k-fold. This may be due to the fact that in the case of k-fold, some segments from certain subjects are included both in the training and testing, which might induce bias from the data leakage problem. From the table, the highest accuracy of 88.58% was obtained with DWT + SuEn + LDA and 21 selected channels of 64. With fewer channels, DWT + TShEn achieved accuracy scores of 87 and 85% using linear SVM and KNN classifiers with 10 and 14 channels, respectively. Of all the previous studies, only two^27,33^ verified their methods using a validation method other than the k-fold CV method. The authors of^27^ used the same dataset (the UNM dataset) and held out two subjects for test and the remaining subjects for training, obtaining an accuracy of 75%. In^33^, with the same dataset, LOSO CV was used, obtaining a classification accuracy of 85.40%. The authors of^33^ also concluded that there is no statistically significant effect of levodopa-ON and levodopa-OFF sessions in the UMN dataset. This conclusion is also supported by the results in Tables 10 and 11, where the classification results of off-PD versus HC did not differ much from the results of on-PD versus HC. Therefore, in the current investigation (LOSO CV), it was sufficient to present the classification results of on-PD versus HC (Table 12).

**Table 12:** Classification accuracy for off-PD versus HC based on leave one subject out CV technique.

FE method + classifier	Accuracy (No. of selected channels)				
	RF	SVM_Linear_	SVM_Quadratic_	LDA	KNN
DWT + LogEn	79.63 (9)	83.95 (8)	80.86 (5)	83.33 (18)	83.95 (9)
DWT + ThEn	78.40 (8)	87.04 (13)	83.64 (9)	87.65 (14)	82.41 (16)
DWT + SuEn	79.63 (9)	83.33 (11)	83.33 (6)	88.58 (21)	83.64 (15)
DWT + TShEn	77.78 (10)	87.04 (10)	84.57 (8)	82.72 (9)	85.19 (14)

## Advantages, limitations, and future studies

As we mentioned earlier, the aim of our study is to provide an efficient and, at the same time, less complex model than those found in previous studies. There is no doubt that deep learning-based models^26–30^ offer promising results, but at the cost of simplicity. For example, the number of trainable parameters reached 100 K in^30^, 20 K in^28^, and 6602 in^26^. The model in study^27^ (CNN + LSTM) used the lowest number of parameters, which was 380. In terms of resource utilization, deep learning techniques need high-performance memory and processors. For example, the model developed in^28^ (CNN + RNN) has been implemented with a GPU machine, as the authors reported. The authors of^29^ stated that their developed model necessitates a large amount of computer memory. The CNN-based model in^26^ was executed on a computer with two Intel Xeon 2.40 GHz processors and a 24 GB random access memory. Regarding the execution time, the authors of^28^ reported that their model required 15 min for training, but once it has been trained, it takes less than a second to test a new segment. The authors of^29^ also reported that their model (2D-CNN) is computationally demanding, which results in long training times. Other studies did not report information related to the execution time. Because the study^30^ developed a 2D-CNN-based model, like^29^, it is expected that the model in^30^ would also require long training times. As the authors of^27^ used fewer parameters, it is expected that their model uses less computational resources compared to other models.

Despite the good performance of deep learning techniques, using traditional machine learning techniques remains a good option in the absence of suitable computational resources. The study^26^, which is the first study to use 1-D CNN for PD detection, reported that the CNN structure is computationally expensive as compared to conventional machine learning techniques. In these techniques, the number of parameters to be selected is much smaller than the number of trainable parameters in deep learning techniques. For example, in the present study, only one or a few parameters are required for KNN, linear/quadratic SVM, and RF classifiers, while no parameters are required for LDA. Table 3 includes the parameters used. Regarding the few parameters of DWT and entropy metrics, we just used the recommended values in^42,46^. Regarding the computational resources, the proposed methods in the current study were carried out using simple resources: an Intel i3-2350 M CPU @ 2.30 GHz, 8.0 GB RAM, and R2013 MATLAB. With these resources, the execution times were computed. The average time required to extract one feature vector from a raw segment (applying filtering, DWT, and entropy) was 0.605 s. The average time required to train the machine learning models (LDA + SVM + KNN) with 545 feature vectors was around 0.5 s while and test them with 61 vectors was 1.45 s (at a rate of 0.024 s per vector).

By contrasting the results of the proposed methods with those of previous studies, the significance of this study can be assessed. Table 13 summarizes the existing state-of-the-art techniques that use the same publicly available PD datasets: SanDiego and UNM. In addition, the used classification type and the classification scores are also included. The proposed methods in the present study are also included in the table with their corresponding results. The main advantages of our methods can be summarized as follows:

- They are less complex, require low execution time, fewer trainable parameters, and don’t require large amounts of memory, making their hardware implementation easier in reality.
- They achieve good classification accuracy as they have been validated using two datasets from two different sources.
- Robust as they have been extensively cross-validated using 10 rounds of ten-fold CV. In addition, the leave-one-subject-out CV has also been used to validate the proposed methods.
- The proposed methods could achieve high accuracy with a small number of channels.
- To the best of our knowledge, we are the first group to present DWT + different entropy measures + machine learning techniques for the detection of PD.

**Table 13:** Summary of PD detection studies that used the same publicly available PD datasets.

References	FE methods	Classifier(s)	Dataset	Classification	Accuracy (%)
^27^	–	CNN + LSTM	UNM	Off-PD versus On-PD	99.2
^29^	Gabor transformation + 2D-CNN		SanDiego	Off-PD vs. HC On-PD vs. HC Off-PD vs. on-PD	99.44 98.84 92.60
^30^	Smoothed pseudo-Wigner Ville distribution + CNN		SanDiego	Off-PD vs. HC On-PD vs. HC	99.84 100.0
^33^	PSD	Hyperplanes	UNM	Off-PD versus HC	85.40
^34^	WT + statistical measures	SVM	SanDiego	Off-PD versus HC On-PD versus HC	96.13 97.65
^35^	CSP + LogEn	KNN	UNM (close/open)	Off-PD versus HC On-PD versus HC Off-PD versus on-PD	98.81/99.01 98.77/98.85 98.73/98.97
		SVM KNN KNN	SanDiego	Off-PD versus HC On-PD versus HC Off-PD versus on-PD	99.41 95.76 97.52
Present study	DWT + TShEn DWT + ThEn DWT + TShEn	SVM KNN SVM	UNM (close/open)	Off-PD versus HC On-PD versus HC Off-PD versus on-PD	99.51/98.62 99.52/99.20 99.39/98.79
	DWT + TShEn DWT + TShEn DWT + ThEn DWT + SuEn DWT + TShEn	KNN	SanDiego	On-PD versus HC Off-PD versus on-PD Off-PD versus HC Off-PD versus HC Off-PD versus HC	94.21 98.84 99.72 99.66 99.89

Although the proposed methods are uncomplicated and perform well, there are some issues that need to be discussed. The first is about EEG channel selection. Although the greedy algorithm used in the present study is an easy and quick way to select EEG channels, this algorithm does not result in an optimal solution. Future work includes the use of a heuristic optimization method that works to produce the optimal solution. In other words, finding the minimum number of channels that yields the maximum classification accuracy. PD detection using a few channels will be more practical and easier to use. The second issue is the high gap between the performance of 10-fold CV (intra-subject classification) and LOSO CV (inter-subject classification). Our results of inter-subject classification are superior to the results in^27,33^. However, more investigation must be done by researchers to reduce this gap by minimizing the dependence of performance of specific models to subject data. In addition, the use of various datasets in these kinds of studies is one of their drawbacks, which makes it unfair to compare the findings of different studies. It should provide a framework for assessing the researchers' suggested methodologies, which may include utilizing open-source datasets. In the present study, two open datasets were employed to compare our findings to those of previous studies that had also used those datasets. The authors also plan to test and confirm the proposed methods on additional brain disorders like autism and Alzheimer's disease.

## Conclusions

This study introduces efficient discrete wavelet transform (DWT)-based methods for detecting PD from resting-state EEG signals. The features are extracted from the wavelet packet-derived reconstructed signals using different entropy measures, namely, log energy entropy, Shannon entropy, threshold entropy, sure entropy, norm entropy, and T-Shannon entropy. We also investigated the impact of DWT coefficients and the brain regions on the classification accuracy. To classify the extracted features, several classification approaches are also being investigated. All these methods were validated using two public datasets (SanDiego and UNM) with on- and off-medication. According to the results, four entropy measures: log energy entropy, threshold entropy, sure entropy, and T-Shannon entropy, lead to high classification accuracy, indicating they are good biomarkers for PD detection. In the case of off-medication PD versus HC classification, DWT + TShEn achieved accuracy scores of 99.89 and 99.51% for the SanDiego and UNM datasets, with KNN and SVM classifiers, respectively. For on-medication PD versus HC, the KNN achieves accuracy scores of 94.21 and 99.52% when the features were extracted using DWT + TShEn and DWT + ThEn, respectively. The results also indicate that features extracted from all DWT coefficients provide high performance. Regarding EEG channel selection, results show that the frontal region channels contribute the most to classification performance compared with other regions. However, using the forward-addition method, it is found that selecting a suitable small number of channels from several regions could improve the classification accuracy.

## Data Availability

Datasets used are online available: SanDiego dataset: https://openneuro.org/datasets/ds002778/versions/1.0.2. UNM dataset (d002): http://predict.cs.unm.edu/downloads.php.
